# Multifocal Gastric Ulcers Caused by Diffuse Large B Cell Lymphoma in a Patient With Significant Weight Loss

**DOI:** 10.1177/2324709616683721

**Published:** 2016-12-01

**Authors:** Mark A. Gromski, Jennifer L. Peng, Jiehao Zhou, Howard C. Masuoka, Attaya Suvannasankha, Suthat Liangpunsakul

**Affiliations:** 1 Indiana University School of Medicine, Indianapolis, IN, USA; 2 Richard L. Roudebush VA Medical Center, Indianapolis, IN, USA

**Keywords:** lymphoma, weight loss, multifocal gastric ulcerations

## Abstract

Primary gastrointestinal (GI) lymphoma is a heterogeneous disease with varied clinical presentations. The stomach is the most common GI site and accounts for 70% to 75% of GI lymphomas. We present a patient with gastric diffuse large B cell lymphoma (DLBCL) who presented with significant weight loss, early satiety, and multifocal ulcerated gastric lesions. Esophagoduodenoscopy should be performed in patients presenting with warning symptoms as in our case. Diagnosis is usually made by endoscopic biopsies. Multiple treatment modalities including surgery, radiotherapy, and chemotherapy have been used. Advancements in endoscopic and pathologic technology decrease turnaround time for diagnosis and treatment initiation, thus reducing the need for surgery. Health care providers should maintain a high level of suspicion and consider gastric DLBCL as part of the differential diagnosis, especially in those with warning symptoms such as weight loss and early satiety with abnormal endoscopic findings.

## Introduction

Primary gastrointestinal (GI) lymphoma is a heterogeneous disease.^1^ Its clinical presentation varies depending on the location of the disease in the GI tract, staging, and histologic subtypes.^1^ The stomach is the most common extranodal site and accounts for 70% to 75% of GI lymphomas.^1,2^ The majority of cases (~90%) with gastric lymphoma are either of the following 2 subtypes: (*a*) extranodal marginal zone B cell lymphoma of *m*ucosa-*a*ssociated *l*ymphoid *t*issue (MALT) or (*b*) diffuse large B cell lymphoma (DLBCL).^1,2^ MALT lymphomas arise from post–germinal center memory B cells and are related to chronic immune reactions driven by infections such as *Helicobacter pylori* or other autoimmune stimuli.^3-6^ DLBCL is the most common histologic subtype of non-Hodgkin disease.^7^ Patients with DLBCL typically present with a rapidly enlarging symptomatic mass, usually manifested as a nodal enlargement in the neck or abdomen. However, extranodal disease is not uncommon.^7,8^ We highlight a patient with gastric DLBCL who presented with significant weight loss and multifocal ulcerated gastric lesions. The objective of this case report is to increase cognizance for the detection of gastric DLBCL.

## Case Report

**Figure 1.**
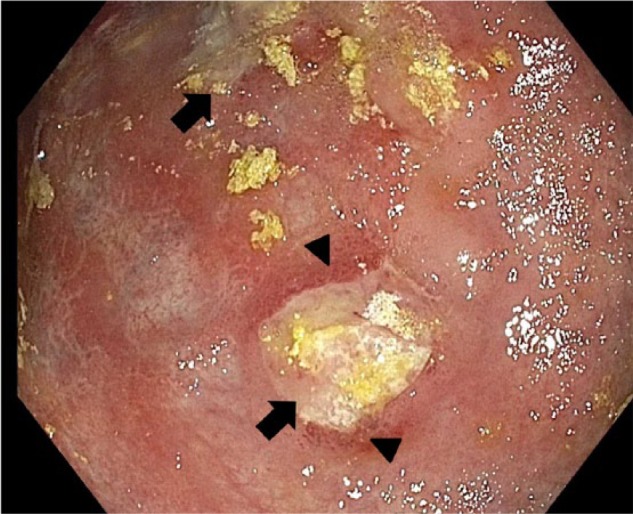
Multifocal ulcerations in gastric body and fundus (arrow), with surrounding diffuse erythema (arrowhead).

**Figure 2.**
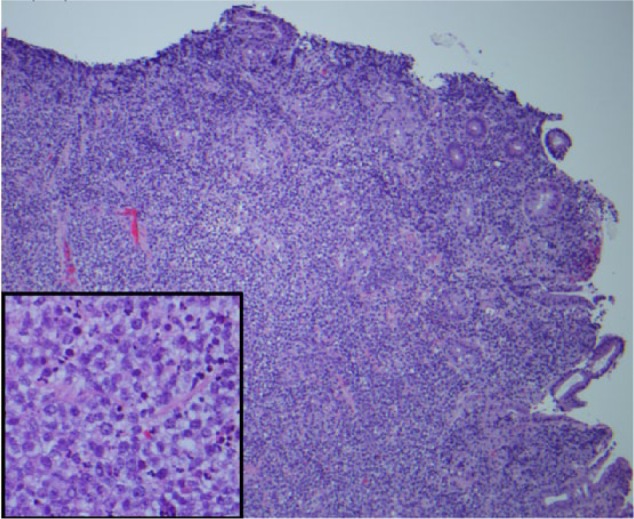
Hematoxylin-eosin section demonstrated ulcerated mucosa with diffuse atypical lymphocytic infiltration effacing gastric architecture. These atypical lymphocytes are large with distinct nucleolus, vesicular chromatin, and increased apoptosis (inset).

**Figure 3.**
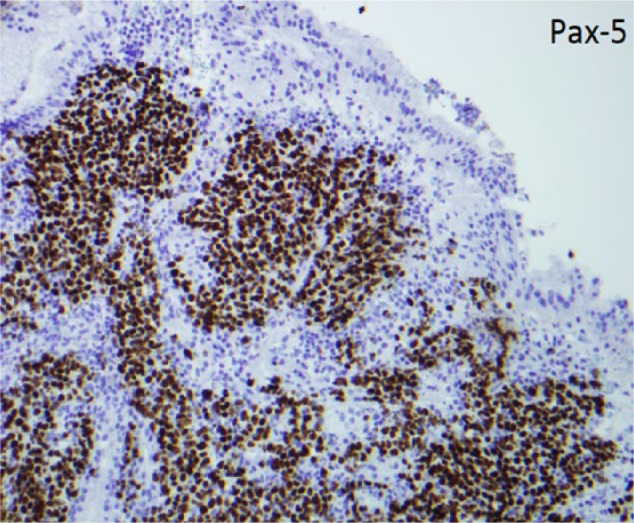
Immunohistochemistry showed that these atypical lymphocytes are positive for Pax-5 (shown) and CD20 (not shown), indicating a B lymphocyte lineage.

**Figure 4.**
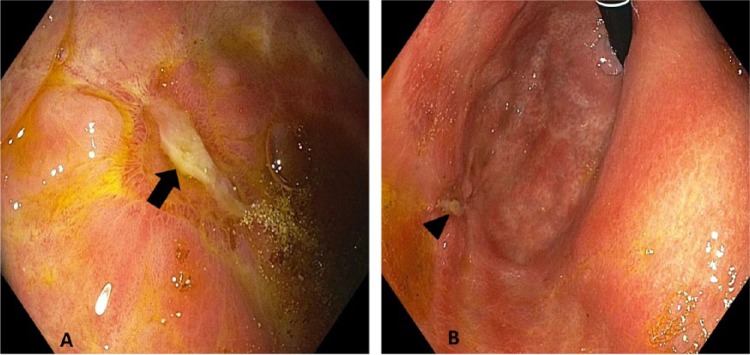
Significantly improved ulceration in the gastric body (arrow; A) and fundus (arrowhead; B).

An 82-year-old male presented with progressive weakness, early satiety, and failure to thrive. He experienced an 80-pound unintentional weight loss in the past 3.5 years, with 34 pounds in the past 8 months. His last colonoscopy 2 years ago showed small tubular adenomas. Physical examination was unremarkable except for cachexia and pallor. Baseline labs were as follows: white blood cell count 4600/µL, hemoglobin 8.5 g/dL, hematocrit 25%, mean corpuscular volume 95.8 fL, platelet count 169 000/µL, blood urea nitrogen 41 mg/dL, creatinine 1.8 mg/dL, total bilirubin 0.24 mg/dL, aspartate transaminase 10 U/L, alanine transaminase 16 U/L, alkaline phosphatase 114 U/L, albumin 3.6 g/dL, total protein 5.8 g/dL, lactate dehydrogenase 118 U/L, iron 92 µg/dL, ferritin 87 ng/mL, iron saturation 35%, positive stool occult blood, folate 8.7 ng/mL, and vitamin B_12_ 490 pg/mL. A chest computed tomography (CT) was unremarkable. An abdominal CT showed mild fat stranding as well as minimal fluid surrounding the ascending colon without any evidence of intraabdominal lymphadenopathy. He underwent an esophagogastroduodenoscopy (EGD) to investigate the cause for significant weight loss and early satiety. EGD demonstrated multiple ulcerated gastric lesions in the fundus and body (Figure 1). The antrum was characterized by mild erythematous gastropathy. Additionally, no sites of active bleeding were found. The ulcerated lesions and the uninvolved antrum/incisura were biopsied. Histopathology revealed ulcerated mucosa with atypical lymphoid infiltrations, characterized by large lymphocytes with irregular nuclei, distinct nucleoli, vesicular chromatin, and evident mitoses and apoptosis (Figure 2). Immunohistochemistry was positive for cluster of differentiation (CD) 45, CD20, paired box 5 (PAX-5; Figure 3), and B-cell lymphoma 6 (BCL-6) and negative for cytokeratin and melanoma associated antigen (mutated) 1 (MUM-1). Ki-67 showed a high proliferative index (80%). The gastric biopsies from the nonulcerated mucosa in the antrum/incisura demonstrated inflammatory cells consistent with gastritis, and immunohistochemistry was negative for *Helicobacter pylori.* The findings are consistent with DLBCL, stage 1E. Gastrectomy was not pursued due to his frailty and advanced age. He was initiated on abbreviated chemotherapy with subsequent involved field radiation. An attenuated immunochemotherapy regimen (R-miniCHOP; rituximab, cyclophosphamide, doxorubicin, vincristine, and prednisone^9^) was started. He tolerated chemotherapy but presented with coffee-ground emesis, after completing 3 cycles of chemotherapy. EGD noted a bleeding ulcer, which was treated endoscopically. There was notable improvement in DLBCL-related ulcers compared to previous EGD (Figure 4A and B). He had no recurrent bleeding and subsequently underwent involved field radiation.

## Discussion

DLBCL is the most common lymphoma, accounting for 25% of all non-Hodgkin cases.^7^ In the elderly (75 years or older), rates of DLBCL increase 1.4% per year.^10^ The molecular pathogenesis of DLBCL is a complex, multistep process that ultimately results in the transformation and expansion of a malignant clone of germinal or post–germinal B cells.^7^ Several genetic changes involving BCL-6 and p53 tumor suppressor genes have been found in DLBCL.^11-13^

Patients with DLBCL typically present with rapidly enlarging lymph nodes in the neck or abdomen. Fever, weight loss, and night sweats are not uncommon and can be observed in up to 30% of patients.^14^ Extranodal disease can occur in up to 40% of cases with the stomach being the most common site.^8^ In general, patients with gastric DLBCL present with nonspecific symptoms, including epigastric discomfort, unexplained weight loss, anorexia, vomiting, and occult bleeding.^1,15^ Hematemesis and melena are uncommon. Additionally, the duration of symptoms preceding the diagnosis is varied.^16^ Diagnosis is usually made by endoscopic biopsies. The endoscopic findings of DLBCL may include mucosal erythema, gastric ulcer, polypoid mass lesion, and/or thickened gastric folds.^17^ It is recommended that both suspicious-appearing as well as normal-appearing mucosa be biopsied since the disease can occasionally present as a multifocal disease with involvement of tissue that appears to be unaffected on initial visualization.^16,18^ Gastric wall thickening can be found on radiographic imaging such as CT scan, though this was not observed in our case.^19^

Histopathology of DLBCL consists of large, transformed B cells with prominent nucleoli, basophilic cytoplasm, a diffuse growth pattern, and a high proliferation fraction (Figure 2).^20^ The immunophenotype of DLBCL can be confirmed by immunohistochemistry or flow cytometry (Figure 3). Gastric lymphoma is less common than gastric cancer but has a more favorable prognosis. Rather than the classic Ann Arbor lymphoma staging criteria, the TNM staging system provides a more accurate prognosis since gastric DLBCL starts locally before spreading to the locoregional lymph nodes prior to distant metastasis, which occurs once the tumor invades into submucosal tissue.^21^ Multiple treatment modalities including surgery, radiotherapy, and chemotherapy have been used. Published reports are based on a limited heterogeneous patient population and, thus, do not allow for comparison of success rates among treatment options. In the past, surgery has been a commonly used modality as it provides rapid symptom control, tissue diagnosis, accurate pathological staging, and can also be curative therapy in patients with early stage disease (IE and IIE),^22^ but associated surgical complications makes it less suitable for frail individuals. Gastrectomy, gastrectomy with adjuvant radiation, and gastrectomy with adjuvant chemotherapy were compared to chemotherapy alone in a small controlled study.^23^ Ten-year overall survival data favored chemotherapy due to the acute and long-term complications of total gastrectomy.^23^ Advancements in endoscopic and pathologic technology decrease turnaround time for diagnosis and treatment initiation, thus reducing the need for gastrectomy. Gastric DLBCL is treated with conventional regimens used for nodal DLBCL, such as R-CHOP,^24^ with excellent response. Attenuated dosage may be required in frail, elderly patients.^9^ Short courses of immune-chemotherapy followed by involved-field radiation is the standard of care for limited stage nodal DLBCL. The 2 approaches, R-CHOP plus radiotherapy and R-CHOP, were compared in gastric DLBCL in a small retrospective study. Three to 4 courses of R-CHOP plus radiotherapy yielded similar 3-year overall survival of over 90%, compared to 6 courses of R-CHOP.^25^ In patients who have already achieved complete remission with immunochemotherapy alone, involved field radiation may not be necessary.^26^ Our patient responded to chemotherapy but still had residual tumors; therefore, radiation was pursued. He achieved pathological remission at the completion of radiation and remains well to date.

## Conclusion

Gastric DLBCL is a rare disease and can present with nonspecific symptoms such as weight loss and early satiety, as in our case. The diagnosis usually requires an EGD with biopsy. Controversies as to standard therapy exist. Chemotherapy and/or immunotherapy, radiation, and surgery are all reasonable choices depending on patient presentation. For early stage disease, surgery is the most rational approach for curative resection, correct staging, and immediate treatment of bleeding or perforations. Adjuvant chemotherapy and radiotherapy are reasonable for advanced stage or residual disease. For stable cases where diagnosis is made by endoscopic biopsy, chemotherapy and radiotherapy can be an alternative primary, noninvasive therapy of choice while surgery is used for residual tumor post chemotherapy/radiotherapy or for treatment of perforation. Chemotherapy and/or radiotherapy is the most rational treatment for stage III and IV disease. Health care providers should maintain a high level of suspicion and consider gastric DLBCL as part of the differential diagnosis, especially in those with warning symptoms such as weight loss and early satiety with abnormal endoscopic findings.
